# Prevalence, inequality and associated factors of overweight/obesity among Bangladeshi adolescents aged 15–19 years

**DOI:** 10.1093/inthealth/ihae012

**Published:** 2024-02-02

**Authors:** Md Sabbir Ahmed, Safayet Khan, Mansura Islam, Md Irteja Islam, Md Musharraf Hossain, Bayezid Khan, Fakir Md Yunus

**Affiliations:** Department of Community Health and Epidemiology, The University of Saskatchewan, 107 Wiggins Road, Saskatoon, SK S7N 5E5, Canada; Department of Development Studies, Daffodil International University, Savar, Dhaka 1216, Bangladesh; BRAC Institute of Educational Development, BRAC University, House 113/A, Road 2, Niketan, Gulshan-1, Dhaka 1212, Bangladesh; School of Educational Studies and Leadership, Faculty of Education, University of Canterbury, Christchurch 8140, New Zealand; School of General Education, BRAC University, Dhaka 1212, Bangladesh; Faculty of Medicine and Health, School of Public Health, The University of Sydney, Sydney, NSW 2006, Australia; Centre for Health Research, The University of Southern Queensland, Toowoomba, QLD 4350, Australia; Research, Innovation and Grants, Spreeha Bangladesh Foundation, Mohammadpur, Dhaka 1207, Bangladesh; Resident Medical Officer (RMO), Upazila Health Complex, Bakshiganj, Jamalpur 2124, Bangladesh; Development Studies Discipline, Social Science School, Khulna University, Khulna 9208, Bangladesh; Department of Psychology and Neuroscience, Dalhousie University, 1355 Oxford Street, Halifax, NS B3H 4R2, Nova Scotia, Canada

**Keywords:** BMI, inequality, nutrition, school, South Asia

## Abstract

**Background:**

The objective of the current study was to estimate the prevalence and associated factors of overweight/obesity among Bangladeshi adolescents aged 15–19 y and to identify whether wealth-related inequality exists for overweight/obesity among Bangladeshi older adolescents.

**Methods:**

We analyzed publicly available national representative secondary data from the 2019–2020 Bangladesh Adolescent Health and Wellbeing Survey. This cross-sectional survey was carried out among 18 249 adolescents aged 15–19 y regardless of their marital status using a two-stage stratified sampling technique (the data of 9128 eligible adolescents were included in this analysis). The WHO reference population for body mass index-for-age (1+Z score) was considered as overweight/obesity.

**Results:**

We found that girls had significantly (p<0.05) higher prevalence of overweight/obesity (11.63%) than boys (8.25%); however, their biological sex as well their age were not significantly associated with higher odds of overweight/obesity. Those who were in their higher grade (grade 11 and higher) in the school and had been exposed to media were more likely (1.67 and 1.39 times, respectively) to be overweight/obesity compared with primary grade (0–5) and those who experienced no media exposure, respectively. Inequality analysis revealed that adolescents belonging to wealthy households had significantly higher rates of overweight/obesity than those in poorer households (concentration index=0.093).

**Conclusions:**

The study exhibited the multifaceted nature of overweight/obesity among Bangladeshi older teenagers, revealing that their school grade, exposure to media content and wealth-related inequality emerged as significant contributing factors. The findings underscore the urgent need for targeted interventions and public health strategies to address the escalating burden of overweight and obesity in this age group.

## Introduction

Overweight/obesity is defined as an unusual or unhealthy accumulation of fat that could be harmful to health.^1^ Worldwide, it has become one of the most challenging health concerns and is linked to more deaths than underweight.^2^ In parallel with the increase in adult obesity, a worrisome obesity rate is notable among children and adolescents. According to the WHO, the global proportion of adolescents with obesity has increased significantly from just 4% in 1975 to >18% in 2016.^1^ Among South Asian countries, the prevalence of adolescent overweight ranged from 11% (in Sri Lanka) to 19% (in India).^3^

Adolescence is a transitional period from childhood to adulthood, during which an individual acquires new behaviors and practices. The dietary practices, food preferences, hygiene routines and lifestyles that contribute to obesity are formed at this age and may persist throughout life.^4^ It has been documented that being overweight or obese in childhood or adolescence is linked to a wide range of medical and psychosocial complications with both immediate and long-term effects.^5^ Among the medical complications, obesity is associated with a higher chance of experiencing breathing difficulties, an increased risk of fractures, hypertension, early markers of cardiovascular disease, insulin resistance and certain types of cancers that may lead to premature death and disability in adulthood.^6^ From the psychosocial point of view, evidence shows associations of weight status with lower self-esteem, poor social relationships and negative experiences in school, including both poor academic achievement and peer problems and disrupted psychological well-being.^7^

Previously, obesity was considered to be a concern for high-income countries only. However, the prevalence of obesity is rapidly increasing in low- and middle-income countries (LMICs), particularly in urban settings.^8^ A systematic review study reported that, from 1998 to 2015, Bangladesh experienced an increased rate of obesity that varied from 1% to 23% among children and from 1.7% to 25.6% among adolescents.^9^ As an LMIC, the primary reasons behind this increased rate of obesity are identified as nutritional, demographic, epidemiological and socioeconomic transitions occurring in this country.^10^ Additionally, youngsters in developing nations like Bangladesh are more likely to consume sugar, salt with a high-fat content, high-calorie meals and micronutrient-poor foods that are less expensive and of less nutritional quality.^11^ These dietary habits, together with a lack of physical exercise, changing modes of transportation, a sedentary lifestyle and behaviors such as watching television and playing video games, low parental education/awareness and family history of obesity, contribute to a significant increase in overweight and obesity.^12^

To the best of our knowledge, the context of obesity and overweight especially among older adolescents remains underexplored in Bangladesh. The aim of this study was to determine the prevalence, wealth-related inequality of overweight/obesity among adolescents in Bangladesh and to identify associated factors of overweight/obesity among adolescents in Bangladesh. Understanding the magnitude of the obesity and overweight problem in Bangladesh and the risk factors associated with such a burden may help in incorporating and/or addressing those risk factors into the existing public health programs, which may eventually contribute to mitigating the escalating prevalence of obesity and overweight in Bangladesh.

## Methods

### Data source

Our present study is based on secondary analysis of publicly available (authorization is required) Bangladesh Adolescent Health and Wellbeing Survey (BAHWS) 2019–2020 data.^13^ The actual survey was a nationally representative cross-sectional survey, and data were collected from ever-married female, unmarried female and unmarried male adolescents aged 15–19 y residing in non-institutional dwelling units. The BAHWS 2019–2020 was conducted by the National Institute of Population, Research and Training (NIPROT) and Ministry of Health and Family Welfare (MOHFW) in collaboration with the International Centre for Diarrhoeal Disease Research, Bangladesh (icddr, b) and MEASURE Evaluation/Data for Impact (D4I). This survey was based on a two-stage stratified sample of households, which involved the sampling of primary sampling units (PSUs) (first stage) and households (second stage). Once the list of PSUs was prepared, PSUs, households and adolescents were selected from each stratum. Detailed protocols of the sampling process, data collection procedure and survey questionnaires are available in the final report of the survey.^13^ A total of 18 249 adolescents were successfully interviewed (response rate 91.7%), of whom 9128 were eligible for anthropometric measurement (height and weight).

### Outcome variable

The outcome variable for this study was ‘overweight/obesity’, calculated based on the body mass index (BMI)-for-age of the survey participants. BMI was calculated as weight/height^2^, where weight was measured in kg and height was initially measured in cm then converted to m. Adolescents whose BMI-for-age was >+1 Z-score of the WHO reference population were considered to be overweight/obese.^14^ The Z-score for age-specific BMI was precalculated in the dataset. Finally, a binary variable was generated coded as 0=not overweight/obese and 1=overweight/obese.

### Independent variable

The demographic and socioeconomic status of the adolescents and their respective households were considered as independent variables for this study. For a two-level multilevel analysis, study variables were grouped as individual- and community-level variables.

Individual-level variables included an adolescent's (i) age (15–17/18–19 y), (ii) sex (female/male), (iii) marital status (ever married/never married), (iv) educational grades (0–5, 6–10 and **≥**11), (v) currently going to school (no/yes), (vi) currently working (no/yes), (vii) media exposure (no/yes) and (viii) household wealth index quintile (lowest, second, middle, fourth and highest).

Community-level variables included (i) area of residence (urban, semi-urban and rural) and (ii) geographical administrative divisions (Barisal, Chittagong, Dhaka, Khulna, Rajshahi, Rangpur, Sylhet and Mymensingh).

### Statistical analysis

Individual datasets were merged and cleaned (i.e. coding, re-coding of variables and case-wise deletion of missing cases) before formal data analysis. To calculate the prevalence of overweight/obesity, descriptive statistics (frequency and percentage) were performed. Pearson's χ^2^ test statistics were performed to distribute the prevalence of overweight/obesity across different independent variables. To identify the factors associated with overweight/obesity, multilevel multivariable regression models were fitted. Because BAHWS data are hierarchical in nature (a two-stage stratified sampling method was followed), multilevel modeling (MLM) was more appropriate over a traditional logistic regression model.^15^ Four separate regression models were fitted for the MLM: Model I was fitted without any independent variables to assess the variance in the outcome variable between clusters (this model is also known as the null model); Model II included individual-level variables only; Model III included community-level variables; and Model IV included both independent- and community-level variables. To measure community variation in the outcome we calculated the intraclass correlation coefficient (ICC), median odd ratio (MOR) and proportional change in variance (PCV).^16^ The ICC value of the null model was >0, which was considered adequate to perform MLM.^15^ Variables that showed p<0.25 in the bivariate analysis were included in the multivariable model, and effects were reported using OR with 95% CI. Akaike's information criterion (AIC) was used to assess the goodness of fit of each model and identify the better fit model. Multicollinearity between independent variables was checked using the variance inflation factor (VIF), and we did not find any multicollinearity in our analysis (VIF<10). To identify wealth-related inequality in overweight/obesity, the concentration index (CI) and concentration curve (CC) approaches were followed, which is a frequently used method in health inequality assessment.^17^ The value of CI ranges between +1 to −1, where a positive value indicates pro-rich inequalities (CC below the 45-degree reference line), a negative value indicates pro-poor inequalities (CC above the 45-degree reference line) and zero indicates no inequalities (45-degree reference line).^18^ Because the outcome variable was binary in nature, Erreygers's correction method was followed to bound the CI value between +1 to −1,^19^ which was also followed in previous research.^17^ All statistical analyses were performed using Stata SE v. 14.2 (StataCorp, College Station, TX, USA) and p<0.05 was considered a significant result. Survey estimates were weighted to ensure the results at the national level.

## Results

The characteristics of our study population are shown in Table 1. Of the total of 9128 adolescents, >63% were aged 15–17 y, 70% were female, about 73% were never married, the majority (78.9%) were in the 6–10 schooling grades and only 16.5% were employed. More than three-quarters (76%) of the sample experienced media exposure and nearly 60% of respondents were from the disadvantaged group (lowest, second and middle quintile combined). The majority (72.1%) were from rural areas and almost one-quarter of the total sample were from the Dhaka division (23.9%).

**Table 1.** Background characteristics of study participants and the distribution of prevalence of overweight/obesity (χ^2^ test statistics)

			Overweight/obese,	p-value
Characteristics	Categories	N (%)	% (95% CI)	p-value
**Individual-level characteristics**				
Age (y)	15–17	5831 (63.88)	10.04 (9.12–11.04)	0.035
	18–19	3297 (36.12)	11.62 (10.35–13.03)	
Sex	Female	6391 (70.01)	11.63 (10.66–12.67)	<0.001
	Male	2737 (29.99)	8.25 (7.03–9.65)	
Marital status	Ever married	2442 (26.75)	15.45 (13.78–17.29)	<0.001
	Never married	6686 (73.25)	8.84 (7.96–9.82)	
Educational grades	0–5	1208 (13.37)	7.39 (5.98–9.10)	<0.001
	6–10	7126 (78.89)	10.71 (9.83–11.67)	
	11 and higher	698 (7.73)	14.86 (12.22–17.97)	
Currently going to school	No	3370 (37.30)	11.29 (10.05–12.65)	0.145
	Yes	5665 (62.70)	10.17 (9.21–11.23)	
Currently working	No	7622 (83.50)	11.25 (10.40–12.16)	<0.001
	Yes	1506 (16.50)	7.38 (5.84–9.29)	
Media exposure	No	2159 (23.65)	7.58 (6.42–8.92)	<0.001
	Yes	6969 (76.35)	11.55 (10.61–12.57)	
Household wealth index quintile	Lowest	1454 (15.93)	5.41 (4.24–6.88)	<0.001
	Second	1962 (21.49)	7.36 (6.10–8.86)	
	Middle	1990 (21.81)	9.19 (7.92–10.65)	
	Fourth	1924 (21.08)	12.65 (10.77–14.80)	
	Highest	1797 (19.69)	17.77 (15.73–20.00)	
**Community-level characteristics**				
Area of residence	Urban	2028 (22.22)	15.66 (13.86–17.64)	<0.001
	Semi-urban	517 (5.67)	8.69 (6.63–11.31)	
	Rural	6582 (72.11)	9.21 (8.27–10.24)	
Division of residence	Barisal	523 (5.73)	9.43 (7.41–11.94)	<0.001
	Chittagong	1745 (19.12)	11.26 (9.50–13.30)	
	Dhaka	2183 (23.91)	13.87 (11.55–16.56)	
	Khulna	875 (9.58)	10.86 (9.18–12.82)	
	Rajshahi	1195 (13.09)	10.76 (9.29–12.44)	
	Rangpur	1113 (12.20)	8.01 (4.75–8.50)	
	Sylhet	680 (7.45)	6.37 (4.75–8.50)	
	Mymensingh	814 (8.92)	7.87 (6.13–10.04)	
**Total**		**9128 (100.00)**	**10.61 (9.79–11.49)**	

Further, bivariate relationships between explanatory variables and overweight/obesity among adolescents are reported in Table 1. All the individual-level characteristics (except whether the adolescents are currently going to school or not) and community-level characteristics included in the bivariate analysis were found to be significantly associated with overweight/obesity (p<0.05 for all).

The fixed effects (measure of association) and the random intercepts for overweight/obesity are demonstrated in Table 2. Model I (the null model) showed that clustering exists in determining overweight/obesity. The ICC of the null model implies about 8.6% of the total variance in the overweight/obesity are attributed to differences between clusters. Table 2 also shows the presence of unexplained cluster heterogenicity, considering the values of the MOR and PCV. For example, the unexplained cluster variation in overweight/obesity was reduced to an MOR of 1.41 in Model IV (when all variables added to the Model I). Furthermore, as portrayed by the PCV, 46.6% of the variance in overweight/obesity across clusters was explained by the individual-level factors in Model II, and 38.8% of the variance in the outcome of interest was attributable to the community-level factors in Model III. Multilevel analysis (Model IV) also revealed that adolescents in higher (**≥**11) grades were 1.67 times (95% CI 1.12 to 2.47) more likely to be overweight/obese compared with their counterparts. Media exposure was found to be significantly (OR 1.39, 95% CI 1.13 to 1.71) associated with overweight/obesity compared with those who did not experience any media exposure. Adolescents with a middle-high household index wealth quintile were more likely to be overweight/obese compared with those with a lowest/second-lower household index wealth quintile.

**Table 2.** Factors associated with overweight/obesity (multilevel modeling)

	Model: I	Model: II	Model: III	Model: IV
Characteristics	OR (95% CI)	OR (95% CI)	OR (95% CI)	OR (95% CI)
**Individual-level characteristics**				
Age (y)				
15–17		Ref.		Ref.
18–19		0.91 (0.77–1.09)		0.91 (0.76–1.08)
Sex				
Female		Ref.		
Male		0.98 (0.79–1.21)		0.99 (0.80–1.22)
Marital status				
Ever married		Ref.		Ref.
Never married		0.44 (0.34–0.56)*		0.44 (0.34–0.57)*
Educational grades				
0–5		Ref.		Ref.
6–10		1.20 (0.91–1.58)		1.25 (0.94–1.65)
11 and higher		1.62 (1.10–2.39)*		1.67 (1.12–2.47)*
Currently going to school				
No		Ref.		Ref.
Yes		1.04 (0.84–1.30)		1.05 (0.83–1.31)
Currently working				
No		Ref.		Ref.
Yes		0.78 (0.59–1.03)		0.76 (0.57–1.01)
Media exposure				
No		Ref.		Ref.
Yes		1.44 (1.17–1.77)*		1.39 (1.13–1.71)*
Household wealth index quintile				
Lowest		Ref.		Ref.
Second		1.26 (0.91–1.75)		1.23 (0.88–1.71)
Middle		1.55 (1.12–2.15)*		1.47 (1.05–2.06)*
Fourth		2.13 (1.53–2.97)*		1.92 (1.35–2.73)*
Highest		3.20 (2.28–4.50)*		2.74 (1.88–3.98)*
**Community-level characteristics**				
Area of residence				
Urban			Ref.	Ref.
Semi-urban			0.49 (0.34–0.70)*	0.53 (0.36–0.76)*
Rural			0.54 (0.45–0.65)*	0.66 (0.55–0.80)*
Division of residence				
Barisal			Ref.	Ref.
Chittagong			0.96 (0.70–1.33)	0.89 (0.66–1.20)
Dhaka			1.40 (0.99–1.97)	1.10 (0.80–1.51)
Khulna			1.17 (0.84–1.62)	0.99 (0.74–1.33)
Rajshahi			1.13 (0.82–1.56)	0.95 (0.71–1.27)
Rangpur			0.86 (0.57–1.30)	0.89 (0.60–1.30)
Sylhet			0.63 (0.42–0.94)*	0.68 (0.46–1.00)
Mymensingh			0.84 (0.57–1.23)	0.85 (0.58–1.23)
Measure of variation				
Variance (SE)	0.309 (0.056)	0.165 (0.044)	0.189 (0.049)	0.133 (0.043)
ICC (%)	8.60	4.79	5.43	3.90
PCV (%)	Ref.	46.60	38.83	56.95
MOR	1.69	1.47	1.51	1.41
Model fit statistics				
AIC	6095.32	5799.63	6046.75	5789.21

Abbreviations: AIC, Akaike's Information Criterion; ICC, intraclass correlation; MOR, median OR; PCV, proportional change in variance; Ref., reference category; SE, standard error.

Model I (null model) was fitted without determinant variables.

Model II was adjusted for individual-level variables only.

Model III was adjusted for community-level variables only.

Model IV (final model) was adjusted for both individual- and community-level variables.

*Significant p-value (<0.05)

**Figure 1.**
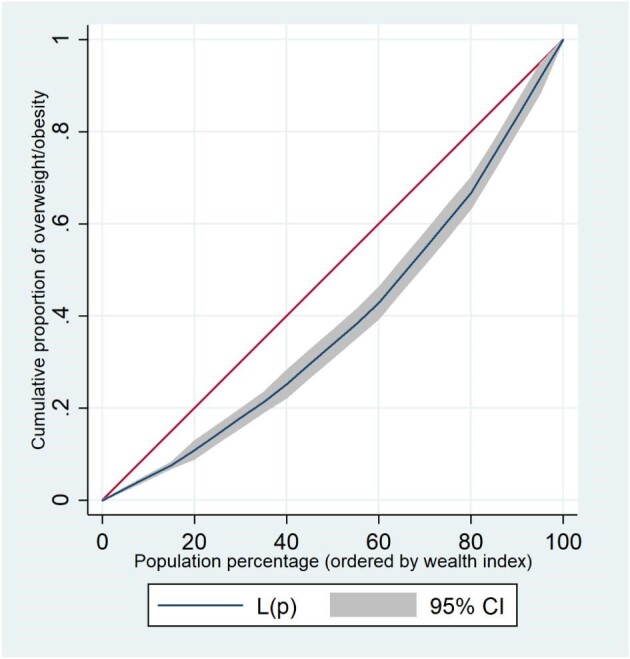
Concentration curve for overweight/obesity. The red line shows the line of equality and ‘CI’ denotes confidence interval. (Concentration Index (SE)=0.093 (0.007), p<0.001).

Figure 1 displays the concentration curve of overweight/obesity, indicating that adolescents (aged 15–19 y) in Bangladesh who live in households with a higher index wealth quintile do experience higher rates of overweight/obesity than those who live in households with a lower index wealth quintile. The overall CI of overweight/obesity in Bangladeshi adolescents was 0.093 (p<0.001).

## Discussion

We investigated the burden of overweight/obesity among Bangladeshi older adolescents and found that >10% of adolescent girls have overweight/obesity, whereas slightly <10% of boys had overweight/obesity. Such an increased prevalence of overweight/obesity may negatively impact not just physical health, but also mental health.^20^ The prevalence of overweight/obesity among adolescents found in this study was lower than in previous statistics in Bangladesh.^21–23^ A nationwide survey might be helpful to better estimate the prevalence. Globally, recent overweight/obesity trends are causing serious public health concern as the number of children and adolescents (aged 5–19 y) with obesity has increased 10-fold in the last four decades.^24^ Several studies have reported the associations between obesity and an increased likelihood of premature onset of chronic illness in later life.^25^ A positive energy balance with higher fat intake, lack of outdoor activities, sedentary activities and fat food culture is associated with the increasing prevalence of overweight/obesity among adolescents.^26^

Our findings have shown that adolescents who are currently studying in higher grades (**≥**11) are more likely to be overweight/obese than those adolescents who are currently in lower grades (6–7). We are not exactly certain of the overweight/obesity of older adolescents due to their school grade (**≥**11), as age did not predict their overweight/obesity. One of the potential reasons could be consumption of the oral contraception pill among adolescent girls in Bangladesh because they get married at a later age of adolescence.^27^ We assumed that such a relationship may exist because our study population was female dominant (70.01%) and we found that the prevalence of overweight among those who were ever married (15.45%) was double that of the never-married population (8.84%). One study reported that the oral contraceptive pill, such as the progesterone-only pill, contributed to the hormonal change in the body that results in overweight/obesity.^28^ Regardless, it is clear that there are gaps in knowledge and awareness regarding healthy dietary practice. Although a rapid increase in late-childhood and adolescent obesity has been found in the USA, China and Brazil,^29^ young-age obesity is not yet considered a major public health problem in Bangladesh.^30^ One research study carried out in Ethiopia reported that early adolescents are 2.45 times more likely to develop obesity than late adolescents.^31^ On the contrary, the likelihood of persistent obesity, as well as the severity of obesity, has been found to increase with age in China, which is consistent with our findings.^32^

In addition to these findings, we found that media exposure predicted adolescents’ overweight/obesity. The relationship between screen media exposure and obesity has been widely researched, and many epidemiological studies revealed positive associations between screen time and obesity. A laboratory-based experimental study revealed that screen media exposure can lead to incremental energy consumption without increased feelings of hunger.^33^ In the context of Bangladesh, it is likely that adolescents are attracted by the fancy junk food advertisements on various media platforms and prefer to consume more energy-dense snacks, drinks and fast food, which makes them prone towards consuming fewer vegetables and fruits.^34^ We further noted that adolescents living in urban areas had a significantly higher prevalence of overweight/obesity compared with those living in rural Bangladesh. Urban residence offers more various social amenities than rural areas, such as access to satellite-connected TV, faster internet that enables access to social media, electricity/power connection and better affordability of junk foods observed via various social media platforms. Our results indicated that those adolescents living in semi-urban or rural areas were less likely to be overweight/obesity than those living in urban areas.

Furthermore, adolescents who are from middle- to high-income households are more likely to be overweight/obese than adolescents from poorer households. There is mixed evidence about the relationship between household wealth and obesity. Although low parental socioeconomic status and overweight/obesity in children is less prominent,^35^ recent evidence from high-income countries has reported an association with overweight/obesity being more prevalent among socioeconomically disadvantaged families.^36^ Similar to our findings, one study revealed that the status of adolescents’ household wealth was significantly associated with obesity, with adolescents in wealthy households 7.03 times more likely to be obese than those in the poorer household wealth quintiles.^31^ Other studies that presented similar findings also discussed the reason, which was the consumption of more energy-dense and protein-rich food by adolescents from high-income households.^37^ It is generally assumed that higher purchasing power may lead to affortability to food that may reduce the undernutrion burden; however, we see that high affortability may also lead to access to unhealthy food. It is likely that older adolescents have their own purchasing power, which may be due to either their different marital status or higher academic grades, where parents are relaxed regarding their outside home food choices.

Not just in Bangladesh, overweight/obesity among children and adolescents remains a global health issue and the literature suggests an increasing prevalence of obesity in LMICs.^38^ Bangladesh is no exception to this recent trend, as in our research we found a high prevalence of obesity among higher secondary students who were from wealthy households and experienced lengthy media exposure. A public health campaign and school-based health promotion strategies are necessary to raise awareness among adolescents about the different risk factors of obesity, and the provision of effective and targeted intervention to the adolescent’s family could lessen the burden of disease caused by obesity.

We acknowledge several limitations to our study findings. Due to the cross-sectional nature of the data analyzed in this study, a temporal relationship cannot be explained with such data. We suggest caution when interpreting these results. Another limitation is that we were unable to consider all possible factors (behavioral, genetic, etc.) in our analysis because of the unavailability of data. However, considering the sampling process, a larger sample size and appropriate analysis approach, the study’s findings can be generalized to adolescents aged 15–19 y in Bangladesh. Future research on adolescent overweight/obesity should consider the use of contraceptives and/or dietary practices.

### Conclusion

More than 10% of Bangladeshi adolescent girls have overweight/obesity, while boys account for <10%. Older adolescents who are in **≥**11 higher grade at school and those who experience media exposure predicted their escalating overweight/obesity prevalence. Our study further noted that wealth-related inequality exists in Bangladesh; teenagers belonging to more wealthy households experienced more overweight/obesity than those from poorer households. Proper nutritional education focusing on healthy dietary behavior may help to reduce the burden of overweight/obesity in Bangladesh. The findings of this study can be a useful resource for policymakers to establish a referral system in the existing healthcare delivery system for adolescents identified at risk, connecting them to nutritionists, counselors and other relevant healthcare professionals.

## Data Availability

Data used in this study are freely available from the UNC Dataverse (https://dataverse.unc.edu/).
